# Nanoplasmonic Paper-Based Platform for General Screening of Biomacromolecules

**DOI:** 10.3390/nano10122335

**Published:** 2020-11-25

**Authors:** Ferran Pujol-Vila, Andrew Tobias Aveling Jenkins, Xavier Muñoz-Berbel, Jordi Mas Gordi

**Affiliations:** 1 Instituto de Microelectrónica de Barcelona (IMB-CNM, CSIC), Bellaterra, 08193 Barcelona, Spain; xavier.munoz@imb-cnm.csic.es; 2 Department of Chemistry, University of Bath, Bath BA2 7AY, UK; chsataj@bath.ac.uk; 3 Department of Genetics and Microbiology, Universitat Autònoma de Barcelona (UAB), Bellaterra, 08193 Barcelona, Spain; jordi.mas@uab.cat

**Keywords:** nanoplasmonic platform, gold nanoparticles, biomolecular coronas, nanocatalytic activity, hygiene screening

## Abstract

Hygiene assessment in industrial and clinical environments is crucial in the prevention of health risks. Current technologies for routine cleanliness evaluation rely on the detection of specific biomolecules, thus requiring more than one test for broad-range screening. Herein, the modulation of the catalytic activity of gold nanoparticles (AuNPs) by biomacromolecules was employed to develop a nanoplasmonic platform for general hygiene screening. AuNPs were immobilized on cellulose paper by simple adsorption. When ferricyanide was dispensed onto the paper, the AuNPs catalysed the ferricyanide’s dissociation, releasing free cyanide ions that dissolved them. The AuNP dissolution produced an intense colour shift detectable with the naked eye. When biomacromolecules (e.g., proteins and polysaccharides) were present, they spontaneously attached to AuNPs, forming a biomolecular corona (biocorona), reducing their catalytic activity until complete suppression when the NPs were fully covered by molecules. The concentration-dependent decrease in the catalytic activity was here used to quantify biomacromolecules and complex samples such as milk, eggs, soy sauce and yeast extract (in 20 min), with detection limits comparable to those of standard methods, i.e., 0.25 µg mL^−1^ for albumin. This nano-enabled technology may be applied as a broad-range (unspecific) alert system for inexpensive cleanliness evaluation, with potential applications in sensitive sectors including productive industries and hospitals.

## 1. Introduction

Hygiene management is critical in environments such as in the food, beverage and pharma industries; clinical settings; and public spaces such as schools, universities or kindergartens, where contamination is becoming a risk for public health. Apart from intensive cleaning and disinfection, many sectors have implemented routine monitoring protocols to ensure the sufficient cleanliness of surfaces and to avoid biological risks. Hygiene levels in healthcare and industrial settings are currently evaluated by means of technologies detecting specific biomolecules as a proxy for organic matter and/or microbial contamination. Adenosine triphosphate (ATP) and protein testing are among the most widespread methods [1,2]. The ATP bioluminescence assay uses the firefly enzyme luciferase to convert ATP into adenosine monophosphate (AMP) with light emission, which is monitored by a luminometer. This approach allows the rapid and sensitive detection of microbial contamination, since ATP is the universal energy transfer molecule of living cells, but is unable to detect non-metabolic contaminants, e.g., viruses. Protein detection at trace levels is achieved thanks to colorimetric assays based on Bradford or Lowry reagents, among others. Although total protein may be considered a good biological indicator, a host of other biomolecules such as lipids and sugars may promote microbial growth in contaminated environments. Unfortunately, their assays are specific to concrete molecules or families and may not reflect the complexity of biological contamination, which may require the combination of various tests. This becomes particularly important considering the current threats of virus epidemics as well as the harmful effects of secreted bio-products such as microbial toxins [3,4]. Thus, the whole risk is impossible to attain with the selective tests developed to date, and new strategies with wider detection spectra are required to obtain a realistic picture of potential contaminants [5].

Nanomaterials, with their unique sensing [6] and catalytic properties [7] widely surpassing those of their bulk counterparts, are positioned as a very powerful alternative to conventional technologies. In this regard, gold nanoparticles (AuNPs) are taking a preferential position for their unmet catalytic activity, being able to catalyse glucose oxidation or ferricyanide dissociation [8,9], as well as their plasmonic properties that confer their suspensions a characteristic red colour. This plasmonic nature has been widely used in lateral flow analysis for the fast detection of biomarkers in a very low-cost and simple paper-based format [10]. Additionally highly reported, the plasmonic peak position shifts after NP aggregation [11]. This property has been employed in biosensing too, for example, in the detection of the proteins through ionically induced aggregation [12]. This detection scheme is based on the fact that, due to their high surface energy, nanosized materials are prone to attracting biomacromolecules, e.g., proteins, that attach on their surface until completely covering them, forming what is called a biomolecular corona or biocorona [13,14,15,16]. This process is mainly driven by electrostatic, van der Waals and hydrogen bonding [17,18]. Protein-coated AuNPs are sterically stabilized and insensitive to changes in the ionic strength of the medium, while naked AuNPs aggregate, changing their optical properties [19]. This assay allows the fast and simple detection of protein content in samples at trace levels. However, aggregation-based methods are restricted to suspended NPs, being unsuitable for detection in solid-state substrates.

The formation of the biocorona has also been reported to reduce the catalytic activity of NPs in the case of proteins, polysacharides [9] and deoxyribonucleic acid (DNA) [20,21]. On the other hand, the presence of small organic molecules such as citrate did not affect nanocatalysis, while polyethylene glycol (PEG) showed a molecular weight-dependent behaviour, i.e., large PEG molecules (>400 Da) inhibited the catalytic activity of AuNPs, but smaller PEG chains did not [9]. Based on that, the modulation of the ferricyanide-dissociation catalytic activity of AuNPs by the formation of the biocorona was here used in the production of a cost-effective nanoplasmonic platform for general hygiene screening. AuNPs were immobilized by drop-casting on cellulose paper, acting as an eco-friendly and low-cost support material, able to pump the sample by capillarity without the need for external instruments [22,23,24]. When incorporating ferricyanide, it is dissociated by AuNPs releasing cyanide ions that dissolve the AuNPs, which lose their characteristic red colour. The presence of biomacromolecules reduces ferricyanide dissociation and NP dissolution in a concentration-dependent manner. This mechanism was evaluated in albumin and agar solutions, as examples of proteins and polysaccharides, and complex samples such as milk, eggs, soy sauce and yeast extract.

## 2. Materials and Methods

### 2.1. Chemical Reagents

Tetrachloroauric acid, sodium citrate tribasic dihydrate, potassium ferrocyanide trihydrate, casein, fructose, sucrose, glycine and L-lysine were purchased from Sigma-Aldrich (St. Louis, MO, USA). Potassium ferricyanide, bovine serum albumin (BSA), sodium chloride and agar were purchased from ITW Reagents (Glenview, IL, USA). BSA-Alexa FluorTM 647 conjugate was purchased from Thermo Fisher Scientific (Waltham, MA, USA). All the chemicals were of analytical grade and were used as received without any purification steps. All the solutions were prepared in deionizedwater unless otherwise stated.

### 2.2. Synthesis and Processing of Gold Nanoparticles

Gold nanoparticles (AuNPs) were produced by the Turkevich–Frens method [25], which is based on the single-phase aqueous reduction of tetrachloroauric acid by sodium citrate, allowing the size- and shape-controlled synthesis of citrate-stabilized AuNPs. A 2.2 mM sodium citrate solution (150 mL) was heated until boiling (15 min) in a thermal magnetic stirrer (600 rpm). Then, 1 mL of 25 mM tetrachloroauric acid was added to the solution. After 5 min of boiling, citrate-stabilized AuNPs of ~10 nm in diameter at a concentration of ~3 × 10^12^ NPs mL^−1^ were obtained and stored at 4 °C until use. The experiments were carried out within a period of 15 days after synthesis since long-term storage could have compromised the stability of the NPs. All the experiments were carried out at room temperature using a concentration of ~2.3 × 10^12^ NPs mL^−1^. The adsorption isotherm of BSA for citrate-stabilized AuNPs was determined with BSA-Alexa FluorTM 647 conjugate. AuNPs were exposed to different concentrations of the fluorescent conjugate for 10 min, followed by centrifugation (13,000 rpm/50 min) and protein quantification in the supernatant. When necessary, unbound proteins were removed by centrifugation (13,000 rpm/50 min) and washing the pellet 3 times with Milli-Q water.

### 2.3. UV-Vis Spectroscopy and Fluorimetry

Optical measurements were performed in transparent 96-well plates by placing 200 µL of sample per well. A Varioskan Flash microplate reader (Thermo Fisher Scientific, Waltham, MA, USA) was used for spectrum acquisition within a range of 350 to 700 nm. The water baseline was always subtracted from the measured spectra. The fluorometric determination of BSA-Alexa FluorTM 647 was performed in black 96-well plates with excitation at 650 nm and emission at 668 nm. All the experiments were carried out in triplicate, and averaged values with the corresponding standard deviations were calculated.

### 2.4. Ferricyanide Dissociation Activity

The catalytic ferricyanide dissociation by AuNPs and subsequent gold dissolution were monitored by UV-vis spectroscopy using a Varioskan Flash microplate reader (Waltham, MA, USA) since the reaction produces a decrease in the plasmonic band at 522 nm until disappearance when cyanidation is completed.

### 2.5. Transmission Electron Microscopy (TEM), Scanning Electron Microscopy (SEM) and Energy-Dispersive X-ray Spectroscopy (EDX)

NPs were imaged with a JEM-2011 transmission electron microscope (ELECMI, Zaragoza, Spain))operating at an accelerating voltage of 200 kV, from the Servei de Microscòpia at Universitat Autònoma de Barcelona (UAB, Bellaterra, Spain). The samples were prepared by drop-casting 5 µL of sample onto carbon-coated copper TEM grids and dried at room temperature. Visualization was performed on various parts of the grids to obtain a global picture of the sample. Size distribution analysis was performed by measuring at least 150 NPs using the free software ImageJ. The plasmonic paper was imaged with a MERLIN FE-SEM operating at an accelerating voltage of 1 kV. EDX analysis was performed with a coupled Oxford LINCA X-Max detector (Oxford Instruments, Abingdon, UK).

### 2.6. Dynamic Light Scattering and Zeta Potential

A Malvern Zetasizer Nano ZS instrument was employed for dynamic light scattering (DLS) (Malvern Panalytical, Malvern, UK) and zeta potential measurements, with a light source wavelength of 532 nm and a scattering angle of 173° for the detection of DLS. Non-adsorbed BSA was removed since free proteins could interfere with the proper characterization of the NPs. All the experiments were carried out in triplicate, and averaged values with the corresponding standard deviations were calculated.

### 2.7. Plasmonic Paper Fabrication

Citrate-stabilized AuNPs were embedded within cellulose matrices (absorbent paper discs, 0.7 mm in thickness) by drop-casting a volume of 100 µL of AuNP suspension and drying at 60 °C until complete dehydration (30 min). After four inoculation/drying cycles, paper matrices containing ~9.2 × 10^11^ NPs were obtained, presenting a deep purple colour owing to the plasmonic nature of AuNPs. The paper strips were then stored at 4 °C until use. For sample analysis, volumes of 100 µL of sample solutions were dropped onto the papers.

### 2.8. Colour Analysis

For colour analysis, images were acquired with a phone camera (13 megapixels) and analyzed using the free software ImageJ (National Institutes of Health (NIH), Bethesda, MD, USA), as reported [26]. Colour images (RGB) were split into the three primary colour channels (red, green and blue). The green channel image, corresponding to the complementary colour of red/purple, was selected for the analysis of the plasmonic colour. The images were converted into grey-scale, and the grey magnitude was found to be inversely proportional to the purple colour intensity. The average grey magnitude of two perpendicular lines crossing the center of the paper discs was obtained, and the corresponding standard deviation was calculated. All the experiments were carried out in triplicate, and averaged values with the corresponding standard deviations were calculated.

## 3. Results and Discussion

**Figure 1:**
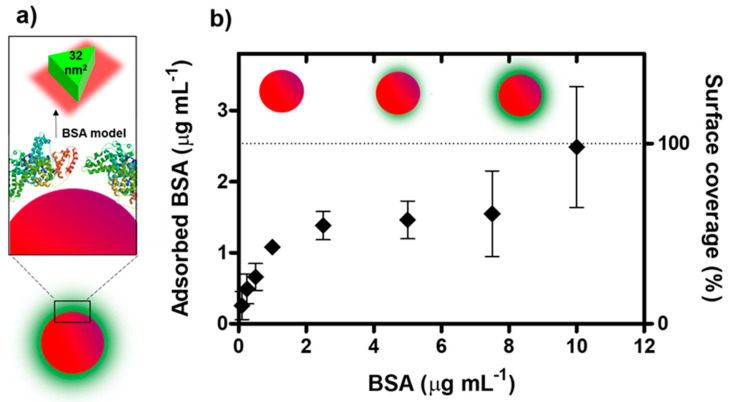
Protein corona formation. (**a**) Illustration of the bovine serum albumin (BSA) corona and BSA molecules approximated by an equilateral triangular prism (not drawn to scale). (**b**) Adsorption isotherm of BSA onto gold nanoparticles (AuNPs) (left y-axis), and estimated percentage of AuNP surface coverage as a function of the BSA concentration (right y-axis) (error bars represent the standard deviation, *n* = 3).

The impact exerted by biomolecular coronas on catalytic ferricyanide dissociation by AuNPs was investigated using citrate-stabilized AuNPs of 10 nm in diameter (illustrated in Figure 1a). Citrate prevented AuNP aggregation by electrostatic forces, although it could be easily replaced by other ligands with stronger affinities, e.g., biomolecules, in the biocorona formation process. Concerning NP size, both the catalytic activity and the capacity for biocorona formation were taken into account. The catalytic activity is inversely proportional to the NP diameter, and hence, smaller NPs present higher catalytic activities [27]. However, small-sized NPs may present dimensions similar to, or even below, those of some common biomacromolecules such as proteins, being unable to develop a full biocorona [28]. Regarding the latter, a size of 10 nm was selected for this study.

### 3.1. Biocorona Formation and Characterization

Bovine serum albumin (BSA), with a size of 66.5 kDa, was used as a model molecule for biocorona formation. Citrate-stabilized AuNPs (~2.3 × 10^12^ NPs mL^−1^) were exposed to fluorescently labelled BSA (BSA-Alexa Fluor) at concentrations ranging from 0.1 to 10 µg mL^−1^ for 10 min, which was enough to reach adsorption equilibrium (Figure S1). The amount of adsorbed proteins was determined by fluorescence analysis after the interpolation of the fluorescent values with the calibration curve obtained by measuring BSA solutions at different concentrations (Figure S2). The adsorption isotherm was then obtained by plotting the amount of adsorbed BSA against the total BSA concentration in the solution (Figure 1b, left *y*-axis). As shown, the amount of adsorbed BSA increased proportionally with the BSA concentration in the solution up to 1 µg mL^−1^, and complete saturation was reached at 10 µg mL^−1^. The experimental data were complemented with a theoretical analysis considering the native conformation of the BSA molecules as equilateral triangular prisms with 8 nm sides and 3.5 nm heights, with triangular cross-sections of 32 nm^2^ (Figure 1a), based on previous publications [29]. The theoretical number of adsorbed proteins to complete a monolayer was estimated by dividing the AuNP surface by the cross-sectional area of BSA, corresponding to ~10 BSA molecules per NP. Extrapolating to the experimental data, where solutions containing 2.3 × 10^12^ NPs mL^−1^ were used, a concentration of adsorbed proteins of ~2.5 µg mL^−1^ should be enough to produce the complete coverage of the NPs (Figure 1b, right *y*-axis). According to the above experimental results, significant coverage of 70% was observed after incubation with 2.5 µg mL^−1^, although it required a higher concentration of 10 µg mL^−1^ to reach 98% coverage. The coverage values were confirmed through hydrodynamic diameter analysis and colloidal stability evaluation (Figure S3). Thus, it may be concluded that, under these experimental conditions, a full BSA corona can be obtained after 10 min of incubation with 10 µg mL^−1^ samples.

### 3.2. Impact of the Biocorona on Ferricyanide Dissociation by AuNPs

AuNPs present the capacity to catalytically dissociate ferricyanide molecules [8], which was employed to detect biomacromolecules through the mechanism illustrated below:

[Fe(CN)_6_]^3−^ = [Fe(CN)_6−n_]^(3−n)−^+ nCN^−^ (1)

4Au + 8CN^−^ + O_2_ + 2H_2_O = 4[Au(CN)_2_]^−^ + 4OH^−^ (2)

**Figure 2:**
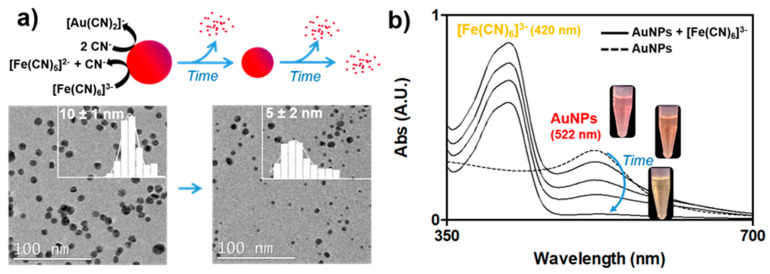
Ferricyanide dissociation by AuNPs. (**a**) Scheme representing ferricyanide dissociation by AuNPs, subsequent gold cyanidation and representative TEM images of AuNPs before and after reaction with 1 mM ferricyanide. Inset, size distribution histograms (the average size was obtained by counting at least 150 NPs). (**b**) Time evolution of the UV-vis spectra of AuNPs with ferricyanide (dashed line corresponds to untreated NPs). Inset, images showing the colour evolution of the AuNP suspension.

First, as shown in Equation (1) (ferricyanide dissociation), the AuNPs dissociated ferricyanide, releasing cyanide ions in the vicinity of the NPs. Then, the free cyanide ions led to the cyanidation of AuNPs as shown in Equation (2) (Elsner equation), producing dicyanoaurate ions as reported in [30,31,32]. The final consequence of the catalytic dissociation of ferricyanide was, therefore, the dissolution and disappearance of AuNPs from the medium, as illustrated in Figure 2. Figure 2a shows the decrease in the AuNPs’ size, from 10 ± 2 to 5 ± 2 nm, after 24 h of incubation in 1 mM ferricyanide (a ferricyanide/gold molar ratio of 8.3) with an NP volume reduction of 80%. It is worth noting that the average final size of the AuNPs was probably overestimated since NPs below 1.5 nm could not be observed and the completely dissolved ones were not considered in this calculation. The NP dissolution entailed a progressive decrease in the plasmonic band of the AuNPs at a wavelength of 522 nm, which completely vanished after 24 h of incubation (Figure 2b). Since no shifts were observed in this band, aggregation processes were disregarded. A second absorbance band at 420 nm was obtained, which corresponded to the yellow-coloured ferricyanide. This band also disappeared over time, although not completely with the excess of ferricyanide in the mixture. The full process could be visually monitored (Figure 2b, inset), where the initial orange solution, resulting from the combination of the red AuNPs with the yellow ferricyanide, progressively became yellow with NP dissolution.

**Figure 3:**
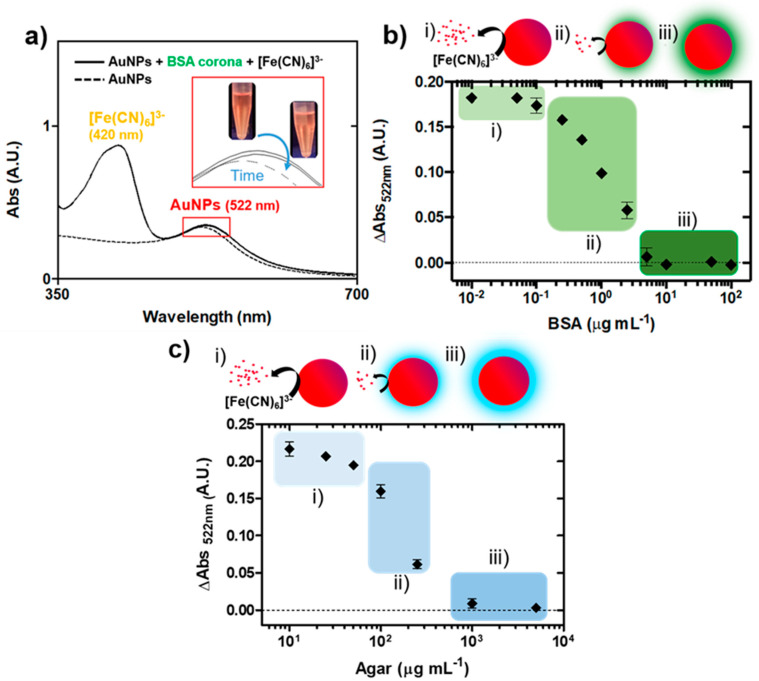
Modulation of ferricyanide dissociation by biocoronas. (**a**) Time evolution of the UV-vis spectra of BSA-coated AuNPs with ferricyanide (dashed line corresponds to untreated NPs). Inset, images showing the colour evolution of the AuNP suspension. (**b**) Absorbance variation at 522 nm of AuNPs (with respect to untreated NPs) exposed to BSA concentrations ranging from 0.01 to 100 µg mL^−1^ after reaction with ferricyanide. The concentration ranges corresponding to low, medium and maximum inhibition of the catalytic activity are indicated by (i), (ii) and (iii), respectively. (**c**) Absorbance variation at 522 nm of AuNPs (with respect to untreated NPs) exposed to agar concentrations ranging from 10 to 5000 µg mL^−1^ after reaction with ferricyanide. The concentration ranges corresponding to low, medium and maximum inhibition of the catalytic activity are indicated by (i), (ii) and (iii), respectively. (error bars represent the standard deviation, *n* = 3).

By contrast, the AuNPs previously coated with a full BSA protein corona (10 min in 10 µg mL^−1^ of BSA) did not catalyse ferricyanide dissociation and remained stable, exhibiting plasmonic bands similar to those of uncoated AuNPs (Figure 3a) and to BSA-coated AuNPs after centrifugation and resuspension in protein-free media with ferricyanide (Figure S4). When the protein corona was not completely formed, the modulation of the catalytic activity was observed, which is illustrated in Figure 3b. BSA concentrations below 0.25 µg mL^−1^ did not modify the catalytic activity of the AuNPs, and the plasmonic peak disappeared after incubation. In the range between 0.25 and 5 µg mL^−1^, a reduction in the catalytic activity proportional to the BSA concentration was found, until complete suppression above 10 µg mL^−1^. This may have derived from a reduction in the NPs’ surface available for catalysis, as already reported for PEG molecules [19]. Considering previous theoretical data, a minimum of 20% coverage (corresponding to the 0.25 µg mL^−1^ concentration) was necessary to produce a detectable reduction in catalytic activity, which progressively and linearly decreased until complete coverage at 10 µg mL^−1^ (full protein corona formation). Similar results were obtained when agar was used as a model polysaccharide molecule instead of proteins (Figure 3c). Thus, polysaccharide coronas, as protein or previously reported DNA ones, modulated the catalytic activity of AuNPs. This provided a simple, fast and sensitive way to detect general biological contamination at trace levels.

**Figure 4:**
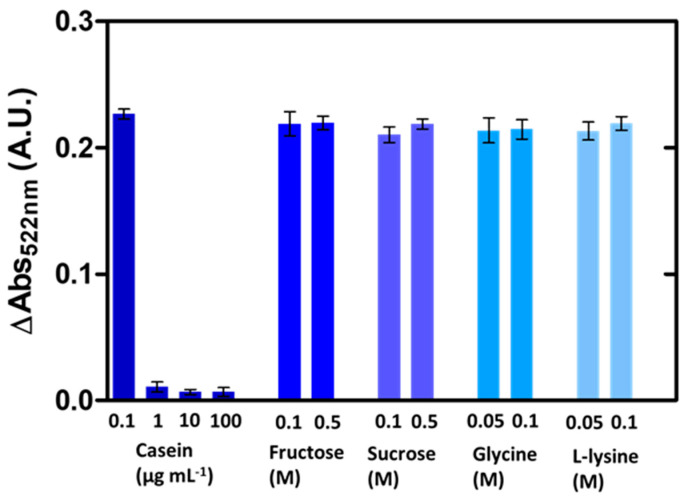
Modulation of the ferricyanide dissociation by small-sized biomolecules. Absorbance variation at 522 nm of AuNPs (with respect to untreated NPs) exposed to casein, fructose, sucrose, glycine and L-lysine after reaction with ferricyanide (error bars represent the standard deviation, *n* = 3).

According to the above results, the biomacromolecules’ adsorption onto the AuNPs’ surface modulates their catalytic activity towards ferricyanide dissociation, and this mechanism may be useful for cleanliness evaluation. Owing to its unpecific nature, this approach may serve as a broad-range alert system for hygiene monitoring in sensitive environments. It is worth noting, however, that the nanocatalytic response may be influenced by both the size and the binding affinity of biomolecules. Therefore, it is difficult to predict the outcome in the case of concrete biomolecules. In this respect, the interference of some typical small-sized biomolecules including sugars (fructose and sucrose) and of aminoacids (glycine and L-lysine) was studied (Figure 4). The catalytic activity of the AuNPs was not affected by the small molecules at the tested concentrations, whereas casein (a typical milk protein (~22 kDa) used as a positive control) induced a decrease at concentrations similar to those observed for BSA.

### 3.3. Nanoplasmonic Paper Platform for General Screening of Biomacromolecules

The AuNP catalysis modulation by biocoronas enabled a sensitive determination of the biomacromolecules in solution. To simplify the assay and the management of residues, AuNPs were deposited on a cellulose matrix (absorbent paper discs) by drop-casting to produce deeply coloured nanoplasmonic platforms with the advantages of being low-cost and presenting capillarity, thus avoiding the use of external pumps for sample/reagent introduction. The optimal amount of NPs in the paper was defined by taking into account two main factors: (i) allowing visual analysis by obtaining deeply coloured papers and (ii) avoiding NP aggregation, since aggregated AuNPs lose their plamonic colour and may lose their catalytic activity as well. Considering this, the use of four inoculation cycles, obtaining papers containing ~9.2 × 10^11^ NPs, was found to be optimal for providing vivid colours without NP aggregation. At higher concentrations of NPs, the papers presented dark colours due to aggregation (data not shown).

**Figure 5:**
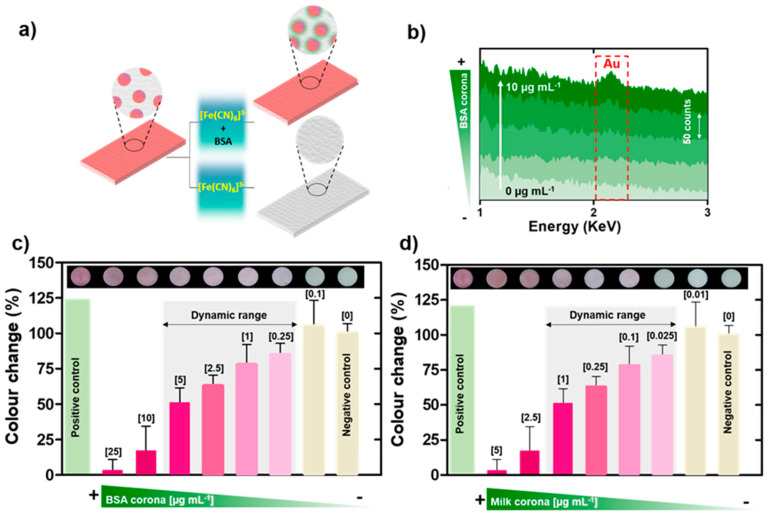
Nanoplasmonic platform for biomacromolecule screening. (**a**) Colour response of the nanoplasmonic paper to biocorona formation. (**b**) EDX analysis of papers exposed to ferricyanide with BSA concentrations ranging from 0 to 10 µg mL^−1^. (**c**) Graph and images showing the colour shift of nanoplasmonic papers exposed to ferricyanide with BSA concentrations ranging from 0.1 to 25 µg mL^−1^ for 20 min. (**d**) Graph and images showing the colour shift of nanoplasmonic papers exposed to ferricyanide with milk concentrations ranging from 0.01 to 25 µg mL^−1^ for 20 min. Colour shift is defined as the degree of change (expressed as percentage) with respect to the positive control (blank nanoplasmonic paper, 0%) and negative control (pristine paper, 100%) (error bars represent the standard deviation, *n* = 3).

BSA was used as a model molecule to validate the platform’s performance. The ferricyanide concentration was increased to 50 mM to avoid diffusion limitations in the cellulose matrix and to ensure fast kinetics for the reaction within 20 min. The nanoplasmonic platform responded to protein concentration similarly to in solution (Figure 5a). That is, the paper completely discoloured after ferricyanide incubation in samples containing below 0.25 µg mL^−1^ of BSA due to AuNP dissolution. The colour intensity progressively increased in the range between 0.25 and 5 µg mL^−1^, while 10 µg mL^−1^ samples almost maintained the initial colour of the paper. SEM and EDX studies confirmed that the colour in these papers after ferricyanide incubation was due to the presence of AuNPs (Figure 5b, Figures S5 and S6), which were not present in the uncoloured paper samples, i.e., those incubated in 0.1 µg mL^−1^ BSA. The visual observations were corroborated by colour analysis using the ImageJ software. After splitting the images in the RGB channels, the green one was selected for being the most sensitive to the purple colour. The degree of colour-shift was calculated with the following expression:

Colour change (%) = (G_T_ − G_P_) × 100/(G_P_ − G_N_) (3)

where G_T_ is the grey magnitude of the test nanoplasmonic paper, G_P_ corresponds to the positive control paper (blank nanoplasmonic paper without biomolecules, 0% change) and G_N_ to the negative control paper (pristine paper without NPs, 100% change).

The changes reported completely coincided with the visual interpretation, validating the possibility of the visual analysis of the samples without the need for external instrumentation (Figure 5c). In any case, the response range (0.25–5 µg mL^−1^) and limit of detection (0.25 µg mL^−1^, empirically determined) of the nanoplasmonic platform were in agreement with other colorimetric protein tests based on non-functionalized gold nanoparticles [12,33] or chemical reagents, e.g., the bicinchoninic acid (BCA) assay, the Bradford assay or the Lowry assay (detection limits = 10–20 µg mL^−1^) [34], which validated the nanoplasmonic platform for protein detection. On the other hand, good sensitivities have also been reported for other etching-based methods applied to the detection of diverse analytes [35,36,37].

**Figure 6:**
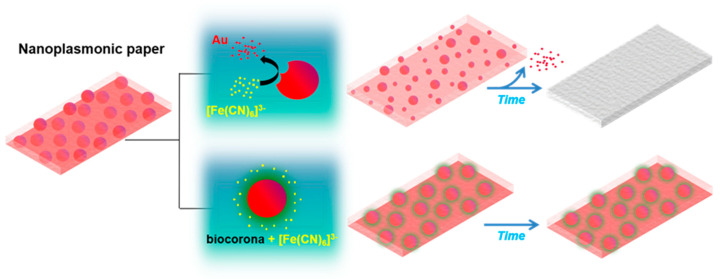
Illustration showing the colour evolution of the nanoplamonic paper in the presence or absence of biomacromolecules forming a biocorona.

**Figure 7:**
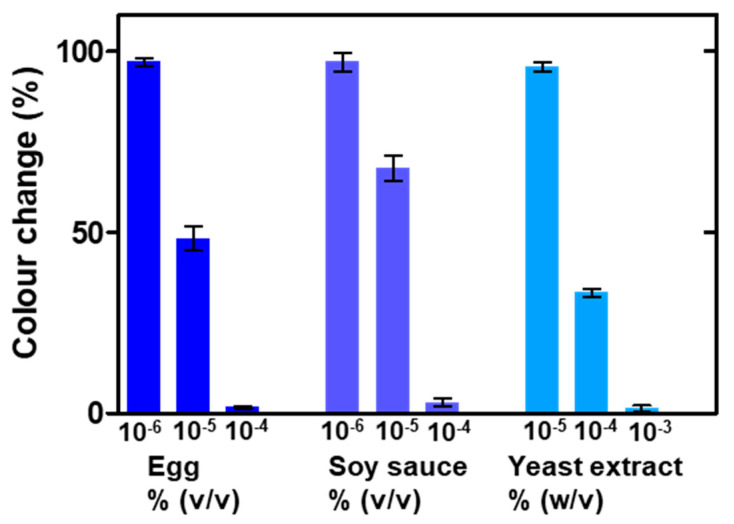
Colour response of the nanoplasmonic paper to different complex samples. Colour shift is defined as the degree of change (expressed as percentage) with respect to the positive control (blank nanoplasmonic paper, 0%) and negative control (pristine paper, 100%) (error bars represent the standard deviation, *n* = 3).

As a step forward, the platform was applied to detect a complex sample. To this end, milk was selected for containing biomolecules of different natures, e.g., proteins, sugars and lipids, and being a well-known allergenic agent causing severe health problems [38]. As shown in Figure 5d, the nanoplasmonic platform presented good sensitivity for whole milk detection, with a detection limit of 0.025 µg mL^−1^ (empirically determined), and a large dynamic linear range of two decades, up to 2.5 µg mL^−1^. The fate of the nanoplatform in the presence or absence of biomolecules is illustrated in Figure 6. These results validated the use of this technology in the determination of contamination from complex matrices such as milk. Additionally, the nanoplasmonic paper’s response to different food samples of different natures was studied to add data supporting the previous conclusion. In this case, chicken eggs, soy sauce and yeast extract (all dissolved in water) were tested with the nanoplatform as before. As depicted in the graphs (Figure 7), all the three samples formed biocoronas, which prevented AuNP dissolution at high concentrations (10^−4^% (*v*/*v*), 10^−4^% (*v*/*v*) and 10^−3^% (*w*/*v*) for the eggs, soy sauce and yeast extract, respectively). At lower concentrations, however, catalytic ferricyanide dissociation and subsequent gold dissolution were not avoided, and thus, colour change was detected. These observations confirmed the suitability of the proposed nanoplasmonic approach for the detection of complex food samples and its potential for general hygiene screening.

## 4. Conclusions

We have developed a low-cost nanoplasmonic platform for sensitive and broad-range hygiene screening based on the modulation of the catalytic activity of AuNPs towards ferricyanide dissociation via biocoronas. Our results indicate that the surface coverage of AuNPs with proteins and polysaccharides, used as model biomacromolecules, modulates ferricyanide dissociation (and subsequent gold dissolution) until complete suppression when a full corona is formed. In the platform, where AuNPs are embedded in a cellulose matrix, AuNP dissolution due to the catalytic activity results in intense colour changes. The formation of the biocorona modulates this catalytic activity, resulting in samples with different colour intensities proportional to the initial biomolecule concentrations, even appreciable with the bare eye. The platform, initially validated with albumin, is capable of providing a rapid (20 min) concentration-dependent response to complex matrices containing proteins, sugars and lipids, such as milk, eggs, soy sauce and yeast extract, with analytical performance comparable to that of standard methods. Considering its unspecific nature, this approach may serve as an alert system for hygiene monitoring in sensitive environments. Hence, broad-range cleanliness evaluation in a user-friendly and inexpensive fashion may be facilitated by this nano-enabled technology.

## Supplementary Materials

The following are available online at https://www.mdpi.com/2079-4991/10/12/2335/s1. Figure S1: Fluorescence kinetics of samples containing AuNPs and BSA-Alexa Fluor^®^, Figure S2: Calibration curve for BSA-Alexa Fluor^®^, Figure S3: Characterization of protein-coated AuNPs, Figure S4: UV-vis spectra of protein-coated AuNPs centrifuged and resuspended in protein-free media after reaction with 1 mM ferricyanide, Figure S5: SEM images of the plasmonic paper, Figure S6: EDX spectra of plasmonic papers exposed to ferricyanide with BSA.
