# Supplementary material for: Nanoplasmonic Paper-Based Platform for General Screening of Biomacromolecules
Source: Nanomaterials (Basel). 2020 Nov 25;10(12):2335. doi: 10.3390/nano10122335 (PMC7760946; doi:10.3390/nano10122335)
Supplement: Supplementary file 1 [file nanomaterials-10-02335-s001.pdf]

Supplementary Materials

Nanoplasmonic Paper-Based Platform for General Screening of Biomacromolecules

Ferran Pujol-Vila [1,*], Andrew Tobias Aveling Jenkins [2], Xavier Muñoz-Berbel [1] and Jordi Mas Gordi [3]

[1] National Center of Microelectronics (IMB-CNM, CSIC), Bellaterra, 08193 Barcelona, Spain; xavier.munoz@imb-cnm.csic.es

[2] Department of Chemistry, University of Bath, BA2 7AY Bath, UK; chsataj@bath.ac.uk

[3] Department of Genetics and Microbiology, Universitat Autònoma de Barcelona (UAB), Bellaterra, 08193 Barcelona, Spain; jordi.mas@uab.cat

* Correspondence: ferran.pujol@imb-cnm.csic.es

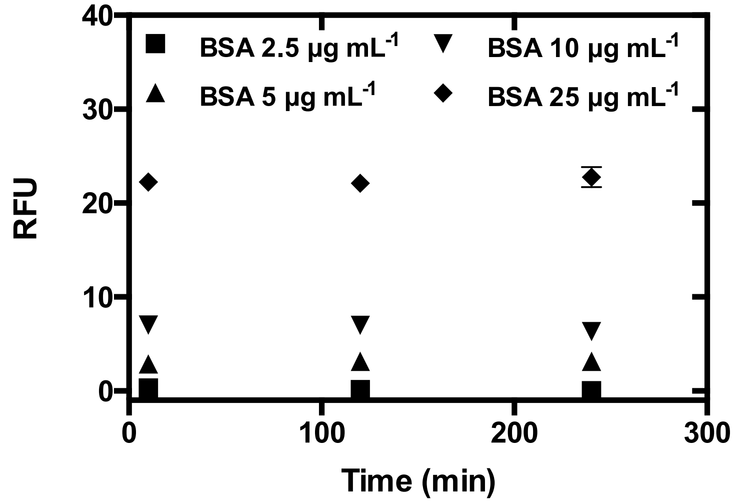

Figure 1. Fluorescence kinetics of samples containing AuNPs and different concentrations of BSA-Alexa Fluor® after removal of adsorbed BSA by centrifugation (error bars represent the standard deviation, n = 3).

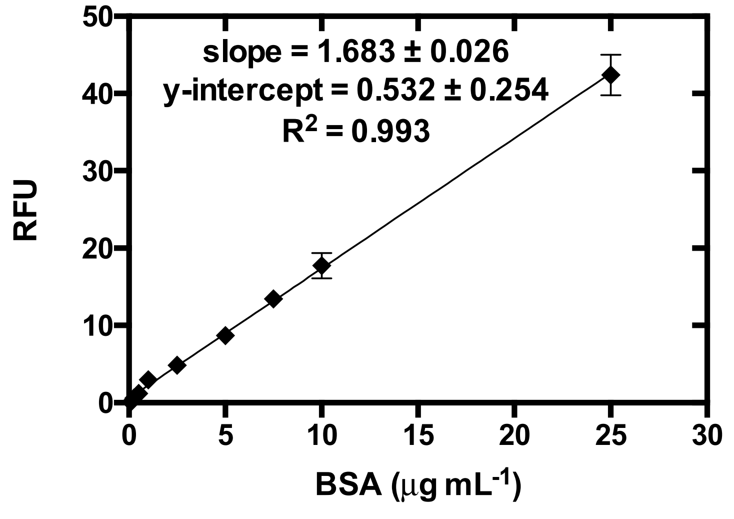

Figure S2. Calibration curve for BSA-Alexa Fluor® fluorescent conjugate. Inset, parameters of the linear fitting (error bars represent the standard deviation, n = 3).

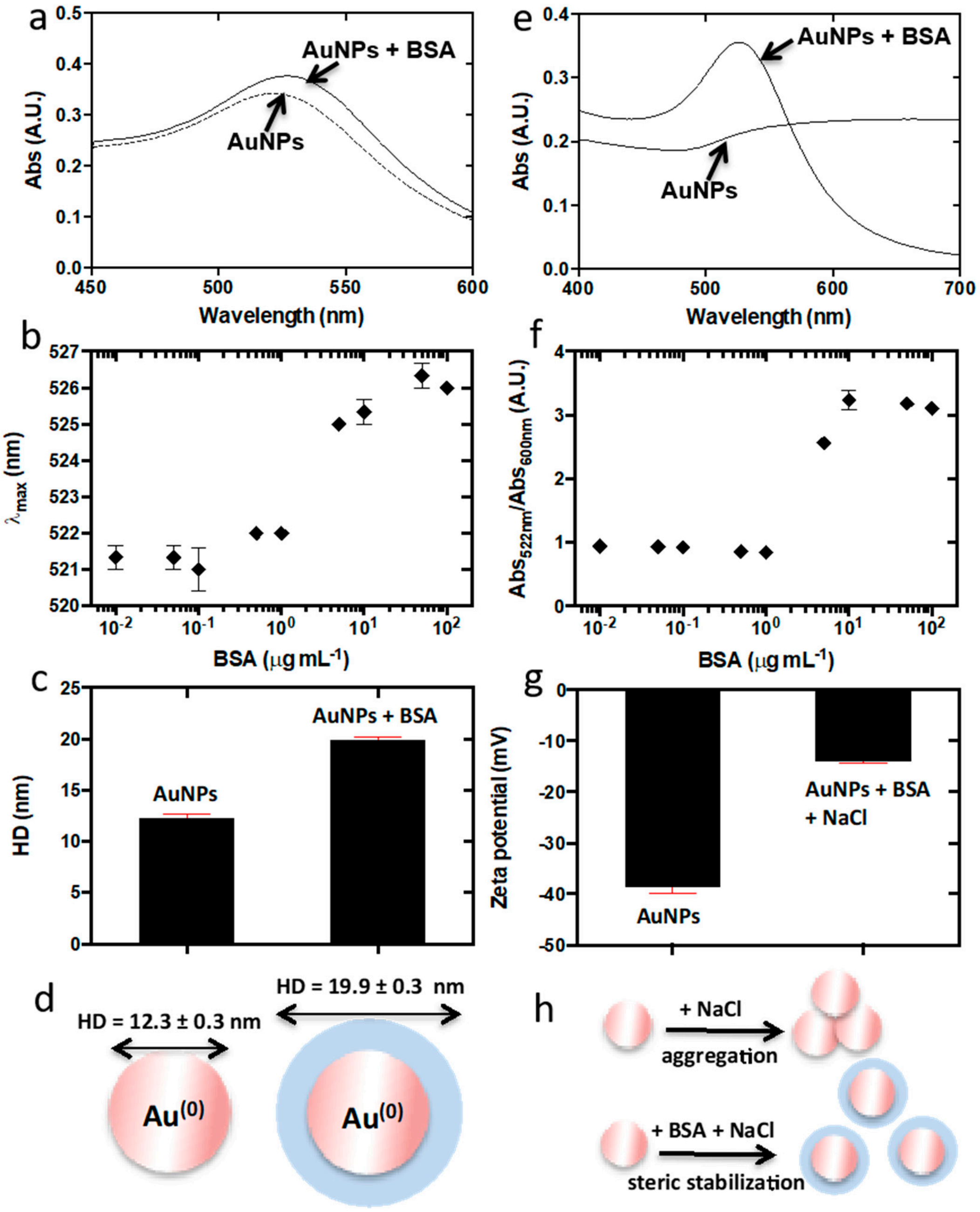

Figure S3. Characterization of protein-coated AuNPs. (a) UV-vis spectra of AuNPs (dashed line) and protein-coated AuNPs (solid line) and (b) maximum absorption (λmax) of the SPR peak of AuNPs exposed to BSA concentrations from 0.01 to 100 μg mL^{-1}. (c) Graph and (d) schematic illustration (not drawn to scale) of the hydrodynamic diameter measured by DLS of AuNPs and protein-coated AuNPs. (e) UV-vis spectra of AuNPs (solid line) and protein-coated AuNPs (dashed line) after addition of sodium chloride to both samples and (f) aggregation factor induced by sodium chloride of AuNPs (i.e. the absorbance ratio between 522 and 600 nm) exposed to BSA concentrations from 0.01 to 100 μg mL^{-1}. (g) Graph showing the zeta potential of AuNPs and protein-coated AuNPs with sodium chloride. (h) Scheme depicting the colloidal stability under high ionic strength of AuNPs and protein-coated AuNPs (not drawn to scale) (error bars represent the standard deviation, n = 3).

The presence and the influence of the protein corona in the AuNPs activity was evaluated by UV-VIS spectroscopy taking benefit of their well-known nanoplasmonic properties. AuNPs were incubated with BSA suspensions from 0.01 to 100 μg mL^{-1} for 5 minutes and analysed spectroscopically. In Figure S3a, the surface plasmon resonance (SPR) peak of AuNPs incubated with 10 μg mL^{-1} BSA is compared to the plasmonic peak of AuNPs solutions without BSA. It is clearly observed how the presence of BSA produced a red-shift of few nanometres in the plasmonic peak, probably due to changes in the resonance energy induced by protein adsorption. This effect was concentration-dependent (Figure S3b). Low BSA concentrations, below 0.1 μg mL^{-1}, did not produce a shift in the plasmonic peak. From 0.1 to 5 μg mL^{-1} BSA, the magnitude of the red-shift was proportional to the BSA concentration, although no changes were observed above 5 μg mL^{-1}. These data were complemented with dynamic light scattering (DLS) measurements, high precision instrumentation that provided the averaged magnitude of the NPs hydrodynamic diameter (HD, in nm). Samples containing AuNPs and AuNPs incubated with 10 μg mL^{-1} BSA were compared. In the second case, AuNPs-BSA samples were centrifuged and resuspended in protein-free media to avoid the interference of non-adsorbed proteins in the measurement. DLS measurements provided a HD of 12.3±0.3 to 19.9±0.3 nm for AuNPs and AuNPs-BSA samples, respectively, with a variation of the HD of 7.6 nm (Figure S3c). This variation could be directly associated to the thickness of the protein corona. The 3.8 nm experimentally obtained as the hydrodynamic radius (1/2 ΔHD) coincided with the height of the BSA molecule (3.5 nm) (Figure S3d). This suggested that the exposition of AuNPs to an excess of BSA (10 μg mL^{-1}) leaded to the formation of a single protein layer.

The colloidal stability of the protein-coated AuNPs was evaluated by inducing aggregation with 0.2 M sodium chloride, which increased the ionic strength of the medium. The stability of citrate-stabilized AuNPs was compared again with AuNPs incubated with BSA. AuNPs samples without BSA showed a red-shift and broadening of the SPR peak until vanishing (Figure S3e). It may be interpreted that the high concentration of counterions shielded the negative surface charge of electrostatically stabilized NPs, allowing intimate contact between them and thus aggregation. In contrast, the SPR peak of protein-coated AuNPs (10 μg mL^{-1} BSA) remained stable, suggesting no aggregation by the steric stabilization of the protein corona. The aggregation factor of AuNPs, defined as the absorbance ratio between the 522 (non-aggregated) and 600 nm (aggregated), was determined and evaluated for the different BSA concentrations (0.01-100 μg mL^{-1}). Results are presented in Figure S3f. From 1 to 10 μg mL^{-1} BSA, there was a concentration-dependent increase in the aggregation factor, which suggested that adsorbed protein was partially preventing AuNPs from aggregation. Above 10 μg mL^{-1} BSA, AuNPs were completely stable and no further increase in the aggregation factor was observed. Plasmonic evaluation of aggregation was complemented with surface charge analysis through zeta potential measurements (Figure S3g). AuNPs were compared with BSA-coated AuNPs exposed to 10 μg mL^{-1}. AuNPs presented a zeta potential of -39±1.2 mV, much higher than BSA-coated AuNPs centrifuged and resuspended in 0.2 M sodium chloride, which presented a zeta potential of -14.0±0.2 mV. It is important to note that the latter, corresponding to the sodium chloride solution, was significantly lower than the value considered the limit for electrostatic colloidal stability, which is around an absolute value of 30 mV. Thus, electrostatic repulsion did not account on the NPs stability under high ionic strength, but steric effects induced by adsorbed proteins may be the main driving force as illustrated in Figure 2h.

These results suggest that a full BSA corona with a thickness of 3.8 nm was obtained by exposition of AuNPs to 10 μg mL^{-1} and this coating induces a red-shift of the plasmonic peak and enhances NPs colloidal stability through steric impediments.

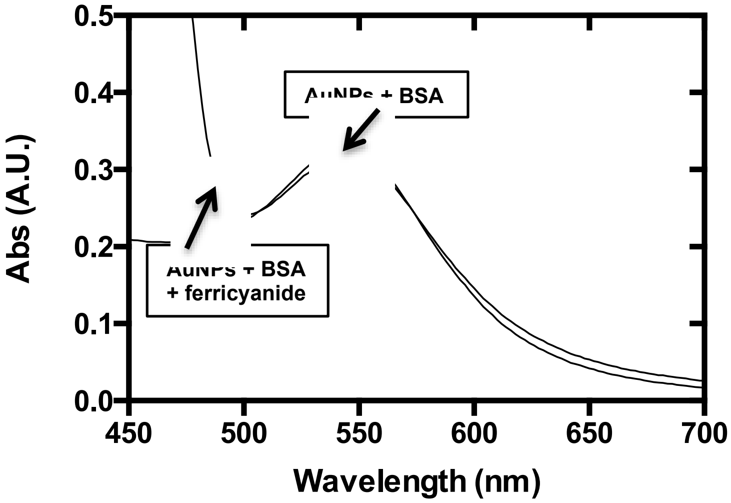

Figure S4. UV-vis spectra of protein-coated AuNPs (exposed to 10 µg mL^{-1} BSA) centrifuged and resuspended in protein-free media after reaction with 1 mM ferricyanide. .

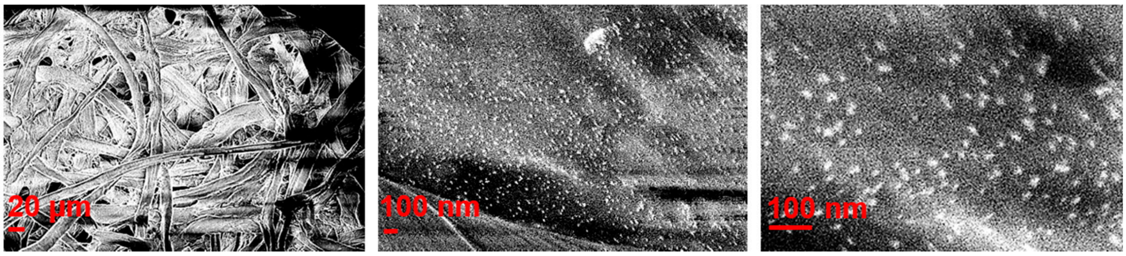

Figure S5. SEM images at different magnifications of the plasmonic paper exposed to solution containing 50 mM ferricyanide and 10 µg mL^{-1} BSA (20 min).

SEM imaging confirmed the presence of AuNPs in the paper strips exposed to high protein concentration (full protein corona) and their complete disappearance in samples without protein. Although presenting a weak purple colour, AuNPs were not observed in the plasmonic papers exposed to BSA from 0.1 to 2.5 µg mL^{-1}. The small size of the partially dissolved AuNPs joined to the charging effects due to the low conductivity of the paper matrices may account for this fact.

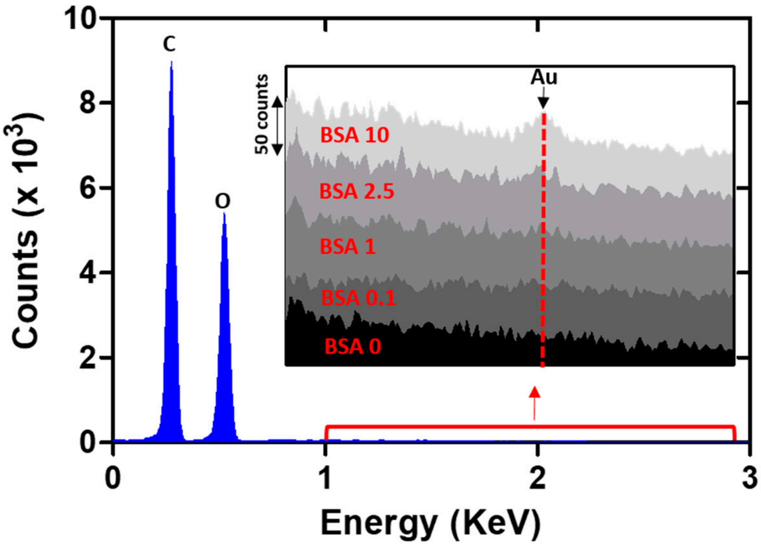

Figure S6. EDX spectra of plasmonic papers exposed to 50 mM ferricyanide with BSA concentrations ranging from 0 to 10 μg mL^{-1} BSA for 20 min. Inset, magnification showing the X-ray emission peak of elemental gold.
